# Involvement of Lysosomal Dysfunction and Its Association With Lipotoxic Stress in Palmitate-Treated HepG2 Cells

**DOI:** 10.7759/cureus.103064

**Published:** 2026-02-05

**Authors:** Susinder Sundaram, Debasree Bishnu, Suman Santra, Swagata Purkait, Debangana Dey, Partha S Mukherjee, Gopal Dhali, Abhijit Chowdhury, Amal Santra

**Affiliations:** 1 Basic Science and Disease Biology, John C. Martin Centre for Liver Research and Innovations, Kolkata, IND; 2 Hepatology, Institute of Post Graduate Medical Education and Research, Kolkata, IND; 3 Public Health, John C. Martin Centre for Liver Research and Innovations, Kolkata, IND; 4 Gastroenterology, Institute of Post Graduate Medical Education and Research, Kolkata, IND

**Keywords:** cell death, lipotoxicity, lysosomal dysfunction, mitochondrial membrane potential, oxidative damage, palmitic acid

## Abstract

Introduction

Nonalcoholic fatty liver disease (NAFLD), characterized by excessive lipid accumulation in the liver, is a growing global health burden. However, the intracellular events contributing to lipid-related liver injury remain incompletely defined. This study investigated the temporal association of lipotoxic events in HepG2 cells exposed to palmitic acid (PA).

Methods

A time course experiment was conducted to evaluate the effects of lipotoxicity on HepG2 cells. Intracellular reactive oxygen species (ROS), lysosomal destabilization, change in mitochondrial membrane potential (MMP), and cell death were assessed using a fluorescence spectrophotometer and a flow cytometer.

Results

Intracellular lipid accumulation and elevated ROS were detected shortly after PA exposure, followed by later alterations in redox balancecompared with control cells. As treatment progressed, significant lysosomal destabilization became evident from 18 hours onwards, leading to the increased cytosolic level and activity of cysteine protease, cathepsin B, into the cytosol. This lysosomal destabilization was associated with mitochondrial dysfunction, assessed by loss of MMP and release of cytochrome c (Cytc) from mitochondria. These events coincided with increased BAX expression, caspase 3 activation, and ultimately resulted in cell death. Importantly, pretreatment with bafilomycin A1 (BAF) markedly attenuated PA-related lysosomal destabilization, prevented cathepsin B activation, and reduced cell death, whereas a contrasting response was observed with chloroquine (CHQ) pretreatment. Together, these findings support a contributory role of lysosomal dysfunction in PA-associated lipotoxic stress.

Conclusion

This study outlines the temporal sequence of organelle dysfunction during PA-associated lipotoxicity and highlights the association of lysosomal impairment in NAFLD pathogenesis. Rather than serving as an upstream trigger, lysosomal impairment appears to function as a late-stage integrator of lipotoxic stress in this in-vitro HepG2 cells model.

## Introduction

Non-alcoholic fatty liver disease (NAFLD) is a prevalent and growing global health issue, primarily characterized by the abnormal accumulation of lipids, particularly triglycerides, in hepatocytes. NAFLD affects 30% of the global population, with a maximum prevalence of 34% seen in South America and the lowest prevalence of 16.1% seen in Australia [1]. The progression of NAFLD can lead to more severe liver damage, including non-alcoholic steatohepatitis (NASH), fibrosis, cirrhosis, and eventually liver failure. Despite the identification of several risk factors such as obesity, insulin resistance, and metabolic syndrome [2], the intracellular events associated with hepatocyte vulnerability in NAFLD remain poorly defined.

Lipotoxicity, which refers to the toxic effects of accumulated fatty acids on cellular components, is a key mechanism of hepatocyte injury in NAFLD. Factors like abnormal lipid metabolism, oxidative damage, mitochondrial dysfunction, endoplasmic reticulum (ER) stress, altered immune responses, gut dysbiosis, and genetic variations in genes contribute towards NAFLD progression [3]. While these processes have been extensively studied, lysosomes have received comparatively less focused attention in the context of hepatocyte lipotoxicity.

Lysosomes are essential organelles that maintain cellular homeostasis through the degradation of lipids, proteins, and other macromolecules. Classically, they play a central role in regulating cell growth and metabolism by supporting autophagy, nutrient sensing, lipid metabolism, and intracellular signaling. Beyond this, some recent evidence has shifted attention toward the involvement of lysosomes in lipotoxicity, where excess saturated free fatty acid hampers their membrane integrity and homeostatic functions [4,5]. Although mitochondrial dysfunction, ER stress, and oxidative damage are well-documented consequences of lipotoxicity [6], lysosomes represent an additional stress-responsive organelle whose role in lipotoxic stress remains incompletely characterized.

In the present study, we address this gap by performing a time-resolved analysis of the response of palmitic acid (PA) on HepG2 cells. Rather than testing a causal hierarchy, the central hypothesis of this work is temporal: that lysosome dysfunction emerges at a defined stage during PA exposure relative to oxidative stress, mitochondrial dysfunction, and apoptotic markers. By mapping the timing of these events, we aim to clarify whether lysosomal changes coincide with, precede, or follow other established features of lipotoxic injury.

## Materials and methods

Materials

The HepG2 cell line was obtained from ATCC (Manassas, VA, USA). Cell culture media, Dulbecco's modified Eagle's medium (DMEM), antibiotics, glutathione (GSH), and malondialdehyde (MDA) detection kits, and most of the chemicals and probes were purchased from Sigma-Aldrich, USA. Fetal bovine serum (FBS) was procured from Gibco, Thermo Fisher Scientific, USA. The PlasmoTest^TM^-Mycoplasma Detection Kit was from InvivoGen, USA. Cathepsin B and D activity assay kits were sourced from BioVision, USA. The cytoplasmic fraction of the cell was extracted using a commercially available kit (G-Biosciences, USA). Except for the caspase 3 antibody, which was from Cell Signaling Technology, USA, all other Western blot antibodies were obtained from Santa Cruz Biotechnology, USA. The Pierce^TM ^ECL Western Blotting Substrate was purchased from Thermo Fisher Scientific, USA.

Cell culture and PA treatment

HepG2 cells were maintained in DMEM supplemented with 10% FBS, 100 U/mL penicillin, and 100 µg/mL streptomycin in a humidified incubator at 37°C with 5% CO_2_. All batches of HepG2 cells used in this study were tested for mycoplasma contamination before any experiment using a commercially available mycoplasma detection kit and were found to be mycoplasma-free.

PA treatment and use of modulators

PA was dissolved in isopropanol at 100 mM and diluted in DMEM containing 1% bovine serum albumin (BSA) to a working concentration of 100 µM. HepG2 cells were treated with or without PA for 1-24 hours to assess intracellular lipid accumulation, intracellular reactive oxygen species (ROS) production, lysosomal dysfunction, mitochondrial dysfunction, and cell death. In some selected experiments, cells were pre-treated with 2 mM N-acetyl cysteine (NAC), a precursor of GSH, or with 100 nM bafilomycin A1 (BAF) or 50 µM chloroquine (CHQ) to modulate lysosomal function prior to PA exposure.

Assessment of intracellular fat by the Nile Red

The cells were fixed with 3.7% paraformaldehyde for 15 minutes at room temperature. To stain intracellular lipid droplets, Nile Red (0.2 mg/ml) was applied and incubated for five minutes at room temperature [7]. Nile Red positive cells were assessed by fluorescence microscope (Zeiss, Axiovert A1, Germany) as well as flow cytometer (BD FACSVerse, USA).

Detection of intracellular ROS

HepG2 cells were treated with or without 100 µM PA and stained with 2.5 µM dihydroethidium (DHE) for superoxide anion (O_2_^−^) detection and 10 µM 2',7'-dichlorodihydrofluorescein diacetate (DCFH-DA) for hydrogen peroxide (H_2_O_2_) detection, incubating for 30 minutes at 37°C in the dark. After staining, cells were trypsinized and resuspended in phosphate-buffered saline (PBS). A fluorescence microscope (Zeiss, Axiovert A1, Germany) was used to detect intracellular ROS, while a fluorescence spectrophotometer (SpectraMax I3x, Molecular Devices, USA) was employed to measure the fluorescence intensity at an excitation wavelength of 488 nm and emission at 535 nm. The results were quantified as arbitrary fluorescence units (AFU). ROS measurement with these probes is interpreted as global intracellular oxidative burden.

GSH assay

The GSH levels in control and PA-treated HepG2 cells were measured using the available GSH assay kit according to the manufacturer’s protocol. Measurements reflect global intracellular redox status.

Lipid peroxidation assay

Lipid peroxidation was assessed in PA-treated and control HepG2 cells using the MDA assay kit according to the manufacturer’s protocol.

Assessment of lysosomal destabilization by the acridine orange (AO) method

Lysosomal stability of cells was measured by AO fluorescence. Cells were incubated with AO solution (5 µg/mL) for 20 minutes in the dark, following the procedure described in our previously published study [8]. AO accumulates in acidic compartments and emits red fluorescence at high intralysosomal concentrations. Changes in AO fluorescence were used to assess lysosomal functional alterations, recognizing that AO redistribution reflects changes in lysosomal acidity or compartmentalization.

Cathepsin activity assay

Cathepsin B and D activities were measured fluorometrically in the cell lysates of control and PA-treated HepG2 cells using the respective assay kits according to the manufacturer's instructions. For the cathepsin B activity assay, fluorescence was measured with an excitation wavelength of 400 nm and an emission wavelength of 505 nm. For the cathepsin D activity assay, fluorescence was measured with an excitation wavelength of 328 nm and an emission wavelength of 460 nm. The results were quantified as AFU. These assays measure overall enzymatic activity.

Mitochondrial membrane potential (MMP) analysis using tetramethyl rhodamine methyl ester (TMRM)

MMP was analyzed in HepG2 cells using TMRM, a membrane-permeable cationic fluorescent dye [9]. HepG2 cells were seeded onto six-well plates and treated with 100 µM PA for varying time periods. Following treatment, the cells were incubated with DMEM containing 50 nM TMRM for 30 minutes. After incubation, cells were trypsinized, washed with PBS to remove excess dye, and analyzed. The fluorescence intensity of TMRM was measured using a fluorescence spectrophotometer (Perkin Elmer FL6500, USA) with excitation at 552 nm and emission at 574 nm. The results were quantified as AFU.

Assessment of cell death

Cell death was assessed in control and PA-treated HepG2 cells using the Annexin-V-FLUOS Staining Kit according to our previously published study [8]. Annexin V detects phosphatidylserine externalization, while propidium iodide (PI) identifies loss of plasma membrane integrity. This assay distinguishes viable, apoptotic, and necrotic populations.

Western blot

At the end of the experiments, cell cytoplasmic extract was prepared using a commercially available kit and subjected to sodium dodecyl sulfate-polyacrylamide gel electrophoresis (SDS-PAGE) on a 12.5% gel to separate proteins based on their molecular weight. The proteins were then transferred onto a polyvinylidene difluoride (PVDF) membrane. Following transfer, the membranes were incubated with primary antibodies targeting cytochrome c (Cytc) (1:300), β-actin (1:1000), caspase 3 (1:200), Bcl2 (1:300), cathepsin B (1:200), p-JNK (1:300), T-JNK (1:300) and BAX (1:300). After washing, the membranes were incubated with appropriate secondary antibodies and protein bands were detected using luminol enhanced chemiluminescence (ECL) reagent. The results were visualized and analyzed to assess protein expression levels.

Statistical analysis

Data are expressed as mean ± standard deviation. Comparisons between two groups were conducted using an unpaired two-tailed Student’s t-test, while analyses involving more than two groups were performed using analysis of variance (ANOVA). For all experiments, p<0.05 was considered statistically significant. All statistical analyses were carried out using GraphPad Prism 8 software (GraphPad Software, San Diego, CA, USA).

## Results

PA exposure is associated with time-dependent intracellular lipid accumulation

**Figure 1:**
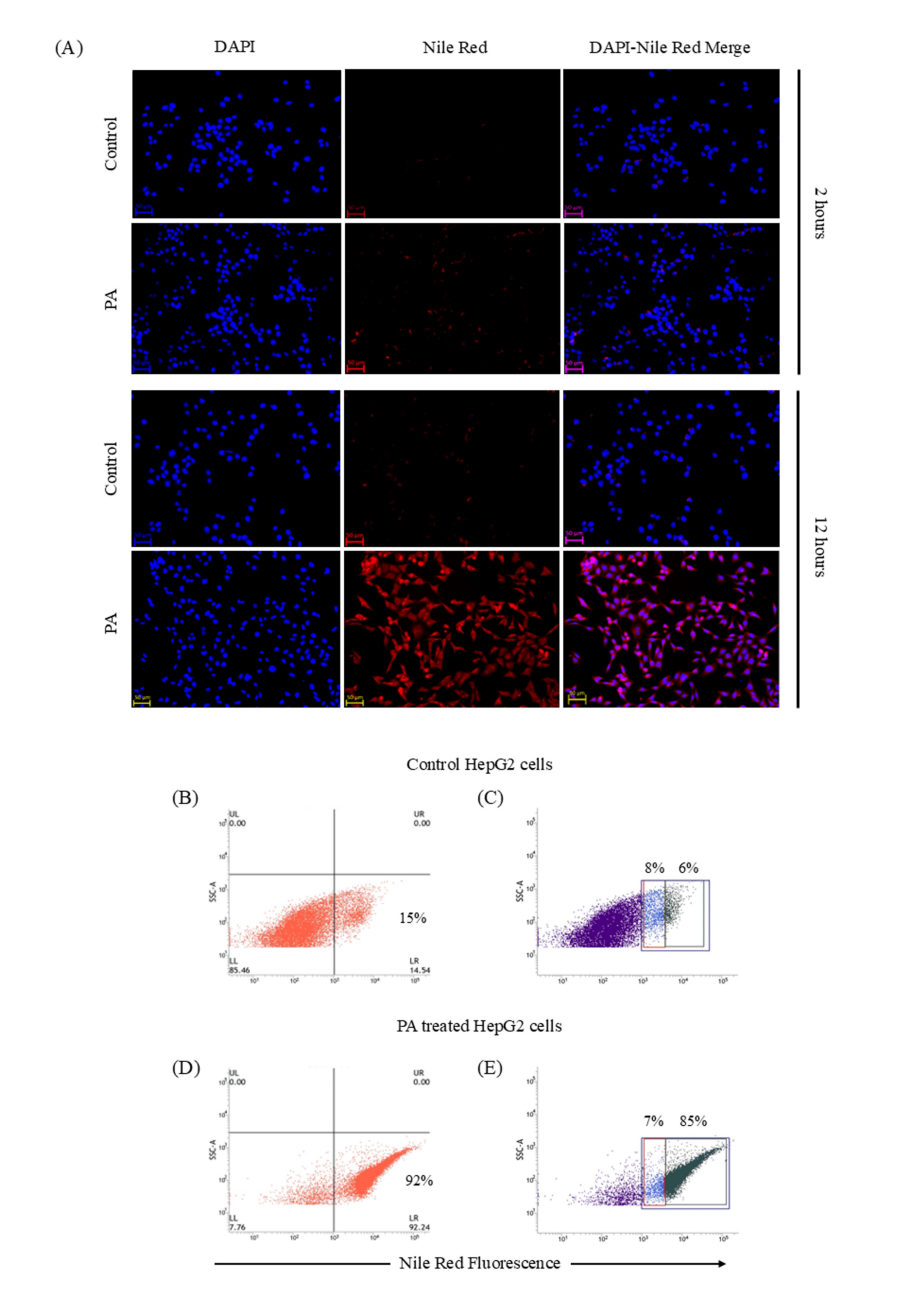
Palmitic acid (PA)-mediated intracellular steatosis in HepG2 cells. Nile red staining was done on HepG2 cells exposed to 100 µM PA. For control, the cells were not exposed to PA. (A) Representative fluorescent photomicrographs (20x) after two hours and 12 hours of PA treatment were shown, respectively (scale bar: 50µm). Cellular steatosis after PA exposure was also evaluated by flow cytometer after staining the cells with Nile red. Dot plots (B) and (C) depicted control HepG2 cells, having 15% cells had detectable fat by flow cytometer, and according to the intensity of Nile red, 8% cells had low fat content, while 6% cells had high fat content. (D) and (E) showed the intracellular fat content of the HepG2 cells, which significantly increased due to treatment with 100 µM PA for 12 hours. DAPI: 4',6-diamidino-2-phenylindole

Following PA treatment, intracellular lipid droplets were detected under a fluorescence microscope after Nile red staining at two hours with a progressive increase parallelled to the time of PA exposure. The majority of cells showed pronounced Nile red fluorescence at 12 hours of fatty acid exposure compared to control cells (Figure 1A). Further confirmation of cellular steatosis was performed by flow cytometry. At 12 hours of PA exposure, approximately 92% of HepG2 cells became Nile red positive (Figure 1D), compared to only 15% in the control group (Figure 1B). Based on fluorescence intensity, Nile red-positive populations were categorized into low and high fluorescence groups. In control cells, only 6% cells displayed high Nile red fluorescence (Figure 1C), whereas PA-treated cells showed a marked increase, with 85% exhibiting high Nile red fluorescence (Figure 1E). These results indicated that PA exposure induced significant lipid accumulation in HepG2 cells. Thus, our present model reflects a state of cellular steatosis, a defining histological feature of NAFLD in humans.

Temporal dynamics of oxidative responses to PA exposure

**Figure 2:**
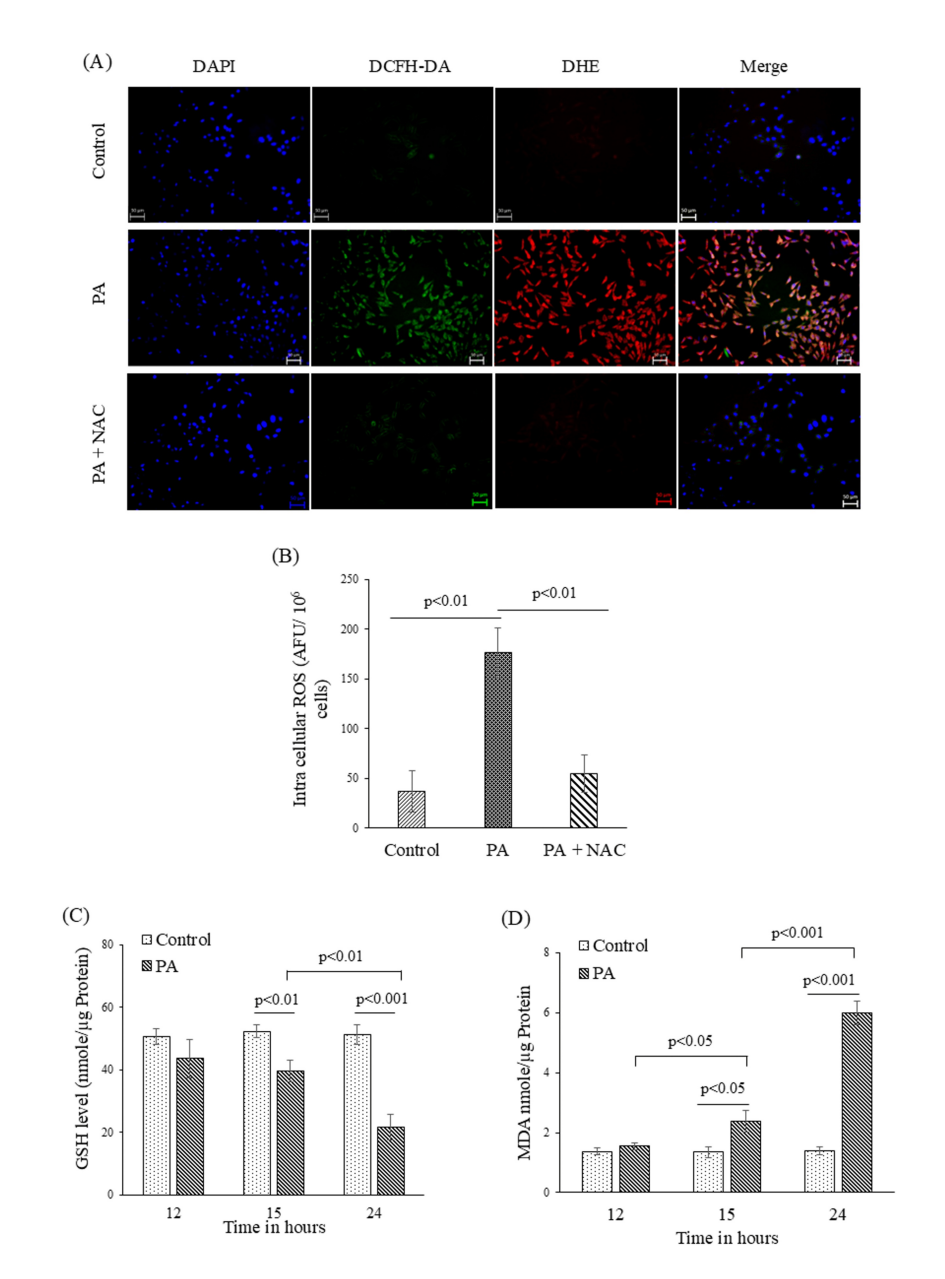
Redox status alterations in HepG2 cells following palmitic acid (PA) exposure. Intracellular ROS was formed in HepG2 cells due to PA (100 µM) treatment. (A) Microscopically, intracellular ROS was evaluated after staining with DHE and DCFH-DA. Nuclei of the cells were stained with DAPI. Visualization of ROS production was carried out using fluorescence microscopy. The representative figure indicated intracellular O_2_^− ^(red fluorescence) and H_2_O_2_ (green fluorescence) generation in HepG2 cells at two hours of PA treatment in the presence and absence of NAC (scale bar: 50µm). (B) Fluorescence spectrophotometric data showing intracellular ROS level per 10^6^ cells in response to two hours of PA treatment in the presence and absence of NAC (n=3). (C) Cellular GSH (n=3) and (D) MDA (n=3) levels were measured respectively in time dependent manner. These bar diagrams represent GSH and MDA levels at 12, 15, and 24 hours of PA treatment by colorimetric assay. Data are presented as mean ± SD, and two-group comparisons were performed using an unpaired two-tailed Student’s t-test. NAC: N-acetyl cysteine; MDA: malondialdehyde; GSH: reduced glutathione; DCFH-DA: 2',7'-dichlorodihydrofluorescein diacetate; DAPI: 4',6-diamidino-2-phenylindole; ROS: reactive oxygen species; DHE: dihydroethidium; AFU: arbitrary fluorescence units

Oxidative stress is a key driver of the initiation and progression of liver injury, and intracellular ROS plays a critical role in this process [10]. In this study, we assessed whether PA-induced steatosis in HepG2 cells was accompanied by elevated ROS production. Intracellular ROS levels were measured using DHE, a probe for O_2_^−^, and DCFH-DA, a probe for H_2_O_2_. Fluorescence microscopy revealed DHE and DCF-positive HepG2 cells within two hours following PA exposure, indicating rapid induction of ROS, which was attenuated by pre-treatment with NAC (Figure 2A).

We further measured intracellular ROS using a fluorescence spectrophotometer. Consistent with the microscopy results, PA treatment for two hours led to a significant increase in ROS production, which was markedly reduced when cells were pretreated with NAC, as evident in Figure 2B. These probes report overall cellular oxidation status, and the observed signal is interpreted as an early oxidative response in the context of PA exposure. In contrast, significant changes in markers of oxidative damage were observed only after prolonged PA exposure. We measured levels of reduced GSH, a major intracellular antioxidant [11], and MDA, a lipid peroxidation marker [12]. Till 12 hours of PA exposure, we did not find any significant changes in GSH or MDA levels compared to control cells (Figures 2C-2D), suggesting that early ROS elevation had not yet translated into detectable oxidative damage or depletion of antioxidant reserves. However, when we measured GSH and MDA levels at 15 and 24 hours time points following PA treatment, we observed significant, time-dependent alterations in their intracellular levels. GSH levels dropped from 15 to 24 hours in PA-treated cells compared to control cells (Figure 2C). Additionally, MDA levels were significantly elevated in PA-treated HepG2 cells at both 15 and 24 hours, in contrast to the control HepG2 cells (Figure 2D).

Lysosomal functional alterations during PA exposure

To assess the impact of PA treatment on the lysosomal status of HepG2 cells, lysosomotropic fluorescence probe AO was used. AO accumulates in acidic lysosomal compartments, and it emits red fluorescence under normal conditions. Alterations in AO fluorescence intensity were interpreted as indicative of lysosomal perturbation, which may reflect changes in lysosomal acidification or functional integrity.

**Figure 3:**
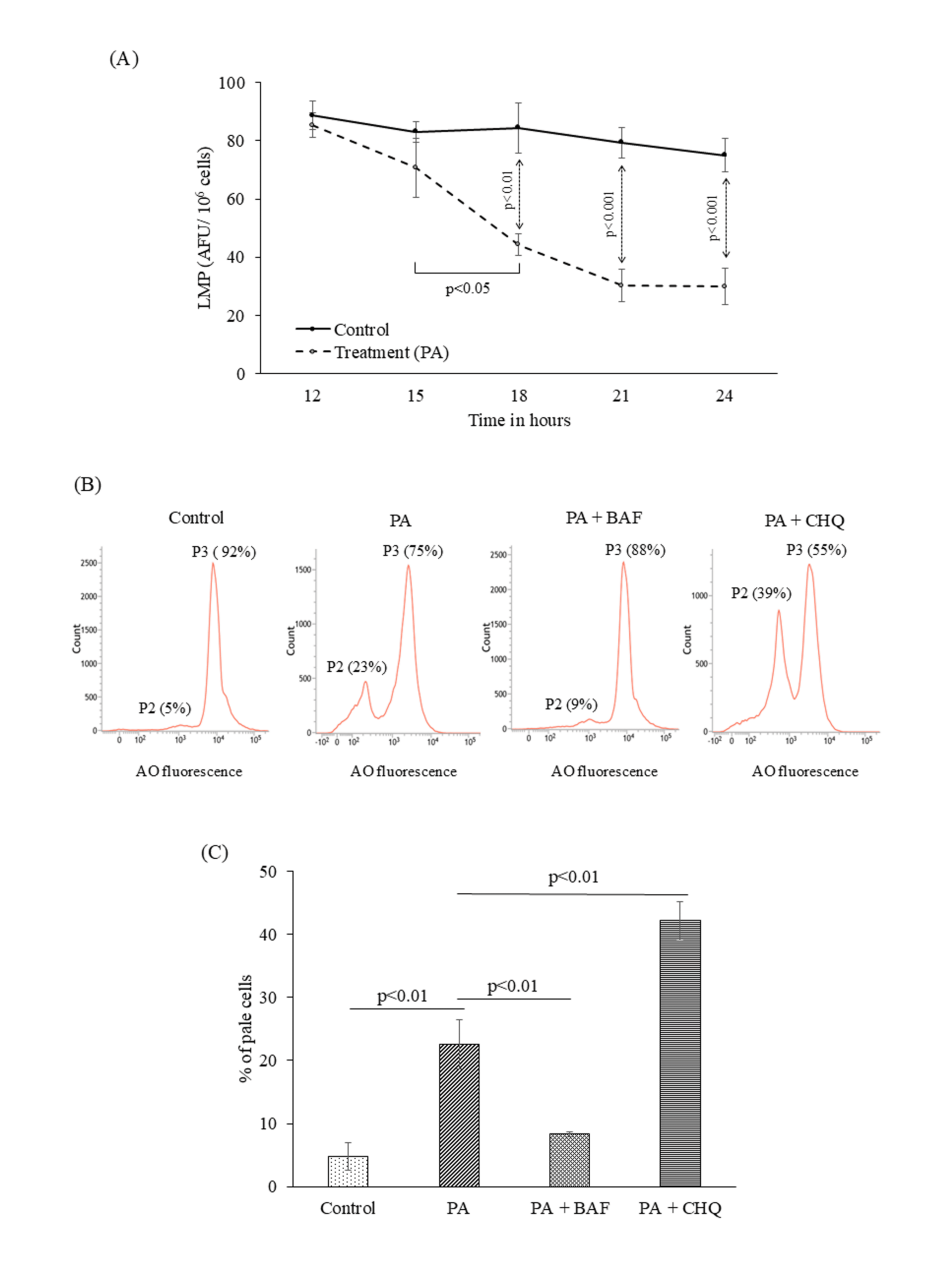
Lysosomal functional perturbation in HepG2 cells following palmitic acid (PA) exposure. Lysosomal functional changes were assessed using the lysosomotropic fluorescent probe acridine orange (AO). (A) Fluorescence spectrometric data show a time-dependent decrease in AO fluorescence from 12 to 24 hours after PA exposure in HepG2 cells (n=3). (B) Histogram plot showing flow cytometric data, depicting the number of cells based on AO fluorescence level in the populations defined in P2 (cells with low AO fluorescence or pale cells) and P3 (cells with low AO fluorescence or bright cells) gates. This figure also represented a change in population percentage of P2 and P3 when treated with PA, as well as in the presence and absence of BAF and CHQ at 18 hours. (C) Bar diagram portrayed flow cytometric data of pale cell percentage when HepG2 cells are treated with PA and compared with those in the presence of BAF and CHQ (n=3). Data are presented as mean ± SD, and two-group comparisons were performed using an unpaired two-tailed Student’s t-test. BAF: bafilomycin A1; CHQ: chloroquine; AFU: arbitrary fluorescence units

We first performed a time kinetics study for the assessment of lysosomal status using a fluorescence spectrophotometer. Till 12 hours of PA exposure, no reduction in AO red fluorescence was observed. However, as the treatment duration increased, a time-dependent reduction in AO red fluorescence was observed beginning at approximately 15 hours of PA exposure, with a significant depletion from 18 hours, as depicted in Figure 3A. This signified a time-dependent deterioration of lysosomal function as the treatment progressed. Based on the time kinetics study, 18 hours was identified as the critical time point at which substantial lysosomal functional loss became evident, a finding further confirmed by flow cytometric assessment. The fraction of "pale cells," which represents low AO fluorescence, denoted by P2 in the histogram (Figure 3B), was significantly increased in HepG2 cells treated with PA for 18 hours. This increase indicates altered lysosomal compartment integrity, highlighting lysosomal destabilization following PA treatment. BAF and CHQ are two pharmacological modulators of lysosomal function [13-15]. Pretreatment of HepG2 cells with BAF prior to PA exposure reduced the proportion of pale cells, whereas CHQ pretreatment increased this population (Figure 3B). The flow cytometric data, represented in Figure 3C, showed that the pale cell population was significantly increased during PA treatment for 18 hours, which was abrogated by pretreating the cells with BAF. Figure 3C also depicted increased population of pale cells in the CHQ pretreated group compared to the PA treatment alone.

**Figure 4:**
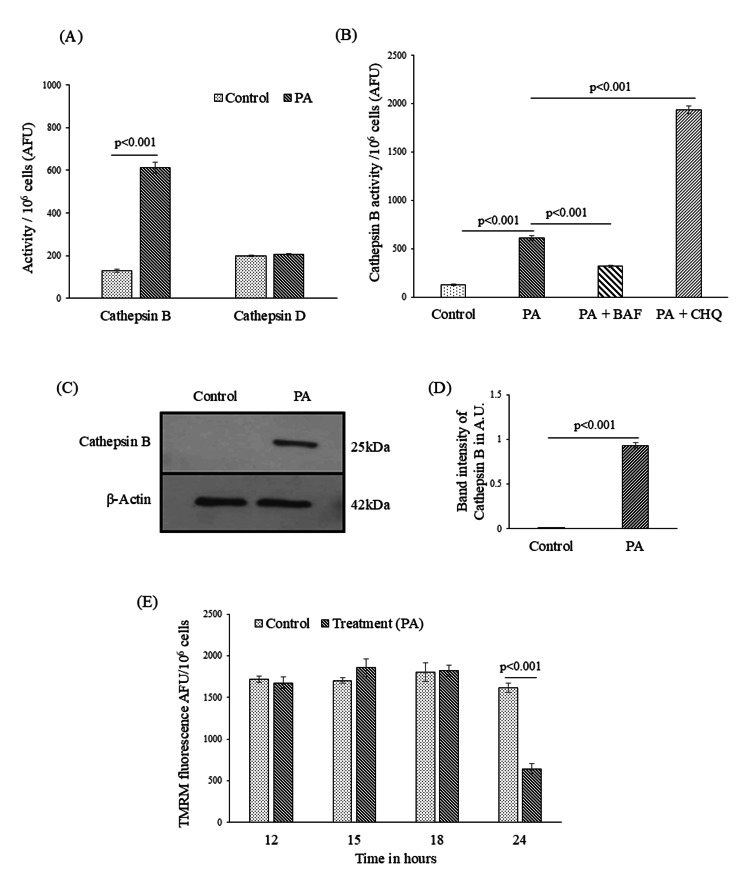
Palmitic acid (PA)-associated changes in lysosomal cathepsin activity and mitochondrial membrane potential in HepG2 cells. (A) Cathepsin B and D activities were measured in whole cell lysates of HepG2 cells treated with or without PA treatment after 24 hours. (n=3). (B) Fluorescence spectrophotometric data showing cathepsin B level in HepG2 cells when treated with PA, PA+ BAF, and PA+CHQ (n=3). (C) Western blot data showing cathepsin B level in the cytosolic fraction in PA-induced HepG2 cells in comparison to control. β-actin was used as a housekeeping gene. (D) The figure depicts Western blot band intensity, which represents protein content of cathepsin B in arbitrary units (A.U.). (E) Fluorescence spectrophotometric assessment of mitochondrial membrane potential in control and PA-treated HepG2 cells, showing emission maxima of TMRM fluorescence at different time points (12, 15, 18, and 24 hours) (n=3). Data are presented as mean ± SD, and two-group comparisons were performed using an unpaired two-tailed Student’s t-test. BAF: bafilomycin A1; CHQ: chloroquine; AFU: arbitrary fluorescence units; TMRM: tetramethyl rhodamine methyl ester

Loss of lysosomal stability can cause the release of lysosomal cysteine protease, cathepsins, and other hydrolases from the lysosomal lumen to the cytosol, which activates death signaling pathways in the cytosol [16]. In the liver, cathepsin B and D are the two most abundant cathepsins released in the cytosol under conditions of lysosomal dysfunction [17]. In this study, we carried out cathepsin B and D activity assay in PA-treated HepG2 cell lysate at 21 hours. Cathepsin B activity in 21 hours PA-treated HepG2 cells was found to be significantly increased compared to control cells (Figure 4A). However, cathepsin D level in the cell lysate did not show any significant change compared to control at 21 hours (Figure 4A).

To confirm the role of lysosomal dysfunction in the release of cathepsin B, we pre-treated the HepG2 cells with BAF and CHQ, 30 minutes prior to PA treatment, to modulate lysosomal function. We observed that cathepsin B activity was significantly decreased due to pre-treatment of BAF, while CHQ pretreatment significantly increased cathepsin B activity (Figure 4B). Western blot analysis of cathepsin B proteins in the cytoplasmic fraction further confirmed its increased expression and release at 21 hours during PA treatment to HepG2 cells (Figures 4C-4D).

Loss of MMP was a late-stage phenomenon that occurred after PA treatment of HepG2 cells

Decrement of MMP is an important feature of mitochondrial dysfunction, which leads to the release of pro-apoptotic factors [18]. We analyzed MMP by a fluorescent spectrophotometer using TMRM at various time points with or without PA treatment. As depicted in Figure 4E, no significant difference in TMRM fluorescence between control and PA-treated HepG2 cells was observed till 18 hours. However, at 24 hours after PA treatment, loss of MMP occurred significantly in the treatment group compared to the control (Figure 4E). These data suggested potential mitochondrial dysfunction at 24 hours of PA treatment.

PA exposure is associated with apoptotic cell death at late time points

**Figure 5:**
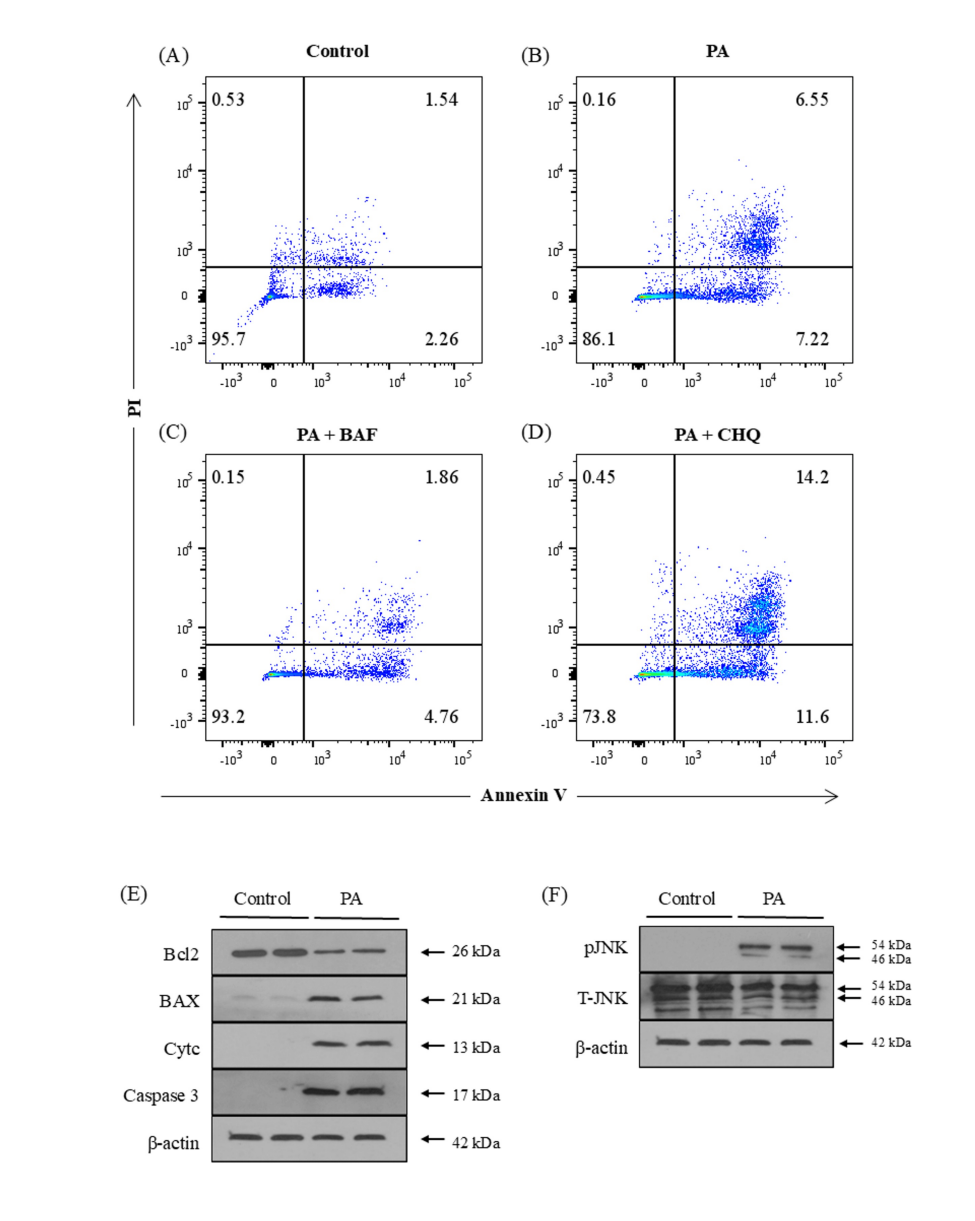
Assessment of cell death and apoptosis-associated markers in HepG2 cells following palmitic acid (PA) exposure. Assessment of cell death of HepG2 cells was carried out using flow cytometry after staining with Annexin V and PI at 24 hours time point of PA treatment compared to control, and represented in quadrant plots. (A) This quadrant plot depicts HepG2 cells without PA treatment, and (B), (C), and (D) showed percentage of dead HepG2 cells by treatment with PA, PA+BAF, and PA+CHQ, respectively. (E) Western blot analysis of anti-apoptotic and pro-apoptotic proteins expressed in the cytosolic fraction of cells with or without PA (100 µM) treatment for 24 hrs. The first two columns represented control HepG2 cells without PA treatment, while the next two columns showed HepG2 cells with 24 hours of PA treatment. In PA-treated cells, anti-apoptotic protein Bcl2 decreased, while the pro-apoptotic proteins (BAX, Cytc, and caspase 3) expression increased in HepG2 cells. (F) This figure represents a Western blot of total and phosphorylated JNK1/2 protein expression in the cytosolic fragment of HepG2 cells with or without PA treatment. In PA treated with HepG2 cells, phosphorylated JNK protein was significantly expressed. β-actin was used as a housekeeping gene. Cytc: cytochrome c; BAF: bafilomycin A1; CHQ: chloroquine; PI: propidium iodide; pJNK: phosphorylated JNK; T-JNK: total JNK

Increased fat accumulation in the liver causes death of parenchymal cells of the liver [19]. In this study, the mode of cell death in HepG2 cells after 24 hours of PA exposure was assessed using flow cytometry following Annexin V and PI staining. The representative quadrant plots in Figure 5 indicated that apoptosis was the predominant form of cell death. In untreated control cells, more than 95% of cells remained viable, with only ~3% undergoing apoptosis (2.48 ± 1.14; n = 3) (Figure 5A). In contrast, PA treatment significantly increased apoptotic cell death to approximately 13% (13.22 ± 1.19; n = 3; p < 0.001) (Figure 5B). Pretreatment with BAF markedly reduced the apoptosis to ~7% (7.79 ± 1.27; n = 3; p < 0.01) (Figure 5C) compared to PA alone. Notably, pretreatment with CHQ further enhanced apoptotic cell death to around 25% (24.22 ± 4.29; n = 3; p < 0.05) (Figure 5D). These findings suggest that lysosomal dysfunction plays an associative role in HepG2 cell death when treated with PA.

We subsequently assessed the expression of apoptosis-associated proteins. Anti-apoptotic protein Bcl-2 acts on mitochondria and prevents the release of Cytc and subsequent caspase activation, as well as the pro-apoptotic protein Bax, which has been shown to accelerate these processes [20]. As depicted in Figure 5E, the Western blot showed a decreased Bcl2 protein and an increase in BAX protein and cytoplasmic Cytc in HepG2 cells at 24 hours of PA treatment but not in control cells, consistent with the findings from the Annexin V and Pl staining evaluated by flow cytometric analysis. To confirm these findings, we also performed a Western blot of caspase 3 in the cytosolic fraction of PA-treated cells and confirmed the increased expression of caspase 3 (Figure 5E). All these data led us to characterize the possible role of mitogen-activated protein kinases (MAPKs) in toxicity during PA exposure. We observed overexpression of phosphorylated JNK after 24 hours of treatment with PA in HepG2 cells (Figure 5F), indicating a potential association between JNK activation and cell death under PA exposure.

## Discussion

Findings of the current paper unfold the temporal progression of subcellular events associated with PA, a saturated fatty acid, in HepG2 cells, and highlight lysosomal destabilization as a distinct stage within the broader lipotoxic stress response. PA exposure in HepG2 cells was associated with a sequence of cellular stress responses that evolved over time, allowing the relative timing of oxidative stress, lysosomal functional changes, MMP alterations, and apoptotic features to be examined without assigning mechanistic hierarchy.

In this study, PA exposure was associated with hepatocellular steatosis detectable within two hours, with a marked increase evident by 12 hours, indicating a clear time-dependent pattern of lipid accumulation. Previous studies have established a close association between cellular steatosis and alterations in cellular redox balance [21]. Consistent with these findings, our results demonstrated a rapid elevation in ROS levels within two hours of PA exposure, preceding significant alterations in redox markers, including GSH and MDA, from 15 hours onward. The absence of oxidative damage during this phase suggests that cellular redox buffering capacity remained largely intact despite elevated ROS levels. This time-dependent pattern suggests that initial oxidative changes reflect adaptive responses to lipid accumulation, while GSH depletion and MDA accumulation emerge with prolonged exposure, indicating disrupted redox homeostasis. PA was selected as the fatty acid used to model lipid-associated cellular perturbations in our cell culture model. The dose of PA, 100 µM, was selected on the basis of previous published work of Malhi et al. [9].

Lysosomes are critical subcellular organelles essential for maintaining cellular homeostasis, and their dysfunction is recognized as a major contributor to cell death pathways. Emerging evidence indicates that lysosomal pH dysregulation may serve as an essential factor in the pathogenesis of liver cell injury [22]. Accordingly, this study examined the involvement of lysosomes in PA-associated hepatocyte lipotoxicity. In the initial hours of PA exposure, in spite of significant intracellular ROS, no cytotoxic effects were detected, and the intracellular lysosomes remained healthy and stable, as evident by high red fluorescence of AO. Lysosomal functional alterations, assessed by acridine orange redistribution, were detected only during these later stages of exposure, as evident by the increased number of pale cell population became apparent from 18 hours of PA treatment. The emergence of lysosomal alterations coincided with oxidative damage markers and preceded or accompanied apoptotic features, placing lysosomal functional impairment within a context of sustained cellular.

Further, a time-dependent loss of lysosomal functional stability was observed, accompanied by activation and cytosolic translocation of cysteine protease, cathepsin B. Modulation of lysosomal function using BAF and CHQ altered both AO redistribution and cathepsin B activity, further supporting an association between lysosomal functional state and cellular stress outcomes.

Mitochondrial dysfunction is a key event in liver injury in different etiologies, including those induced by lipotoxicity [23]. A defining feature of mitochondrial impairment is the change in MMP, which was accompanied by the release of pro-apoptotic factors such as Cytc and alterations in apoptotic signaling. MMP remained largely preserved until late exposure periods, with significant depolarization observed only at the final time point examined, indicating that mitochondrial dysfunction represents a late-stage event in PA-associated lipotoxicity. This timing places mitochondrial functional decline after lysosomal functional alterations and oxidative damage. Consistent with this pattern, changes in Bcl-2 family protein expression, cytosolic Cytc levels, activated caspase-3 content, and apoptotic cell populations were confined to late stages of exposure.

The involvement of JNK in regulating both the intrinsic and extrinsic apoptotic pathways is well established [24]. Phosphorylated JNK not only participates in the upstream activation of pro-apoptotic factors but can also directly enhance their functional activity. The elevated levels of p-JNK observed at 24 hours of PA treatment are therefore consistent with its recognized role in amplifying apoptotic signaling.

Collectively, the temporal pattern observed across these measurements supports a model in which hepatocyte lipid loading is accompanied by early ROS generation. With continued exposure to PA, redox imbalance, lysosomal functional impairment, mitochondrial depolarization, and apoptosis emerge in close temporal proximity. Within this framework, the lysosome appears as a stress-sensitive organelle, with functional deterioration occurring as one of the late-stage events that precedes mitochondrial impairment and apoptosis.

This study had certain limitations. The findings were based solely on in vitro experiments using HepG2 cells, which may not fully represent in vivo liver physiology, and a fixed PA concentration, and acute exposure conditions limit extrapolation to physiological or early disease states. Additionally, only lysosomal functional alteration was examined, and a more detailed analysis of lysosomal function and other lysosomal pathways was not performed. Further in vivo studies and deeper lysosomal investigations are needed to strengthen future studies. Moreover, the assays employed report functional changes at the cellular level but do not provide spatial resolution sufficient to establish causal relationships between subcellular compartments.

In summary, this study outlines how lysosomal function changes over time as part of the broader cellular response to sustained lipid overload. The findings suggest that lysosomal impairment emerges at later stages of lipotoxic stress, along with oxidative damage, mitochondrial dysfunction, and apoptosis. By focusing on timing rather than causality, this work provides a cautious and structured basis for future studies aimed at clarifying event hierarchy and physiological relevance using more detailed and targeted experimental approaches.

## Conclusions

This study defines the timing of cellular changes associated with prolonged PA exposure in HepG2 cells. Early ROS production occurred without detectable oxidative damage, while lysosomal functional alterations, redox imbalance, mitochondrial membrane depolarization, and apoptosis appeared only at later time points. Lysosomal changes coincided with broader cellular stress and modulated the extent of apoptotic cell death. These findings position lysosomal functional impairment as a late-stage feature of lipotoxic stress and provide a temporal framework for future mechanistic studies.

## References

[1] Amini-Salehi E, Letafatkar N, Norouzi N (2024). Global prevalence of nonalcoholic fatty liver disease: an updated review meta-analysis comprising a population of 78 million from 38 countries. Arch Med Res.

[2] Anstee QM, Targher G, Day CP (2013). Progression of NAFLD to diabetes mellitus, cardiovascular disease or cirrhosis. Nat Rev Gastroenterol Hepatol.

[3] Takaki A, Kawai D, Yamamoto K (2013). Multiple hits, including oxidative stress, as pathogenesis and treatment target in non-alcoholic steatohepatitis (NASH). Int J Mol Sci.

[4] Pu J (2022). Targeting the lysosome: mechanisms and treatments for nonalcoholic fatty liver disease. J Cell Biochem.

[5] Zeng J, Acin-Perez R, Assali EA (2023). Restoration of lysosomal acidification rescues autophagy and metabolic dysfunction in non-alcoholic fatty liver disease. Nat Commun.

[6] Chen Z, Tian R, She Z, Cai J, Li H (2020). Role of oxidative stress in the pathogenesis of nonalcoholic fatty liver disease. Free Radic Biol Med.

[7] Malhi H, Bronk SF, Werneburg NW, Gores GJ (2006). Free fatty acids induce JNK-dependent hepatocyte lipoapoptosis. J Biol Chem.

[8] Santra A, Bishnu D, Santra S, Ghatak S, Mukherjee PS, Dhali GK, Chowdhury A (2022). Arsenic-induced injury of mouse hepatocytes through lysosome and mitochondria: an in vitro study. Int J Hepatol.

[9] Sakamuru S, Attene-Ramos MS, Xia M (2016). Mitochondrial membrane potential assay. Methods Mol Biol.

[10] Li S, Tan HY, Wang N, Zhang ZJ, Lao L, Wong CW, Feng Y (2015). The role of oxidative stress and antioxidants in liver diseases. Int J Mol Sci.

[11] Jozefczak M, Remans T, Vangronsveld J, Cuypers A (2012). Glutathione is a key player in metal-induced oxidative stress defenses. Int J Mol Sci.

[12] Janero DR (1990). Malondialdehyde and thiobarbituric acid-reactivity as diagnostic indices of lipid peroxidation and peroxidative tissue injury. Free Radic Biol Med.

[13] Klionsky DJ, Elazar Z, Seglen PO, Rubinsztein DC (2008). Does bafilomycin A1 block the fusion of autophagosomes with lysosomes?. Autophagy.

[14] Pivtoraiko VN, Harrington AJ, Mader BJ (2010). Low-dose bafilomycin attenuates neuronal cell death associated with autophagy-lysosome pathway dysfunction. J Neurochem.

[15] Geng Y, Kohli L, Klocke BJ, Roth KA (2010). Chloroquine-induced autophagic vacuole accumulation and cell death in glioma cells is p53 independent. Neuro Oncol.

[16] Kavčič N, Pegan K, Turk B (2017). Lysosomes in programmed cell death pathways: from initiators to amplifiers. Biol Chem.

[17] Ruiz-Blázquez P, Pistorio V, Fernández-Fernández M, Moles A (2021). The multifaceted role of cathepsins in liver disease. J Hepatol.

[18] Ricci JE, Waterhouse N, Green DR (2003). Mitochondrial functions during cell death, a complex (I-V) dilemma. Cell Death Differ.

[19] Trauner M, Arrese M, Wagner M (2010). Fatty liver and lipotoxicity. Biochim Biophys Acta.

[20] Ola MS, Nawaz M, Ahsan H (2011). Role of Bcl-2 family proteins and caspases in the regulation of apoptosis. Mol Cell Biochem.

[21] Arroyave-Ospina JC, Wu Z, Geng Y, Moshage H (2021). Role of oxidative stress in the pathogenesis of non-alcoholic fatty liver disease: implications for prevention and therapy. Antioxidants (Basel).

[22] Wattiaux R, Wattiaux-de Coninck S, Thirion J, Gasingirwa MC, Jadot M (2007). Lysosomes and Fas-mediated liver cell death. Biochem J.

[23] Di Ciaula A, Passarella S, Shanmugam H, Noviello M, Bonfrate L, Wang DQ, Portincasa P (2021). Nonalcoholic fatty liver disease (NAFLD). Mitochondria as players and targets of therapies?. Int J Mol Sci.

[24] Dhanasekaran DN, Reddy EP (2008). JNK signaling in apoptosis. Oncogene.

